# Combination Gold-Faced Platinum Crown with Porcelain Cusp and Face

**Published:** 1907-06-15

**Authors:** Stanton A. Stuart

**Affiliations:** Cincinnati, O.


### Combination Gold-Faced Platinum Crown with Porcelain Cusp and Face.

BY STANTON A. STUART, CINCINNATI, O.

Tooth to be devitalized, and the stump prepared in the following manner: Cut away the lingual face and shorten the length of crown, and disk off the mesial and destal surfaces. Upon this stump is constructed a band of crown metal or gold-faced platinum, No. 28-g. The band is cut to conform to the ground tooth, and extending a little above outline. A piece of same material, No. 31-g., is now formed to act as floor, and soldered with pure gold; this gives you a cup-like form. A labial facing is now ground to fit, and pins bent to engage the bottom of cup. This is now waxed to place, invested and soldered with pure gold. This cup is then filled in with "Jenkins' Prosthetic" porcelain to fulfill requirements, making an occlusal surface of porcelain, and attaching the facing to the metal cup like solder in the all-gold crown.

*Dummy for Bridge-work.* A suitable facing being selected, it is backed with gold-faced platinum, No. 28-g. which is to be bent at right angles to the surface of the facing so as to form a shelf the labis-lingual width of the crown, and of the same form as a cross section would be. A piece of same material is now formed and soldered to top of the shelf with pure gold to form a cup. This is now placed upon facing and pins bent. It is then filled in with Jenkins' Prosthetic Porcelain to form cusp, according to the requirements.

*Bicuspid Crown for Raising Bite.* Crown made of gold-faced platinum and porcelain.

Make band of gold-faced platinum, No. 28g, letting it extend about one-sixteenth of an inch above the tooth. Cut a piece of same material, No. 31g., to fit inside band, and resting upon upper surface of tooth, solder with pure gold. Now fill in with Jenkins' Prosthetic porcelain to form cusp, according to requirements. This crown can be used on lower molars and bicuspids in cases requiring (raising bite), also where a full upper denture is worn, in cases of trouble from gold cusps being worn through by plate settling.

